# Carotid Artery Intima-Media Thickness, Carotid Plaque and Coronary Heart Disease and Stroke in Chinese

**DOI:** 10.1371/journal.pone.0003435

**Published:** 2008-10-17

**Authors:** Kuo-Liong Chien, Ta-Chen Su, Jiann-Shing Jeng, Hsiu-Ching Hsu, Wei-Tien Chang, Ming-Fong Chen, Yuan-Teh Lee, Frank B. Hu

**Affiliations:** 1 Institute of Preventive Medicine, College of Public Health, National Taiwan University, Taipei, Taiwan; 2 Department of Internal Medicine, National Taiwan University Hospital, Taipei, Taiwan; 3 Department of Neurology, National Taiwan University Hospital, Taipei, Taiwan; 4 Department of Emergency Medicine, National Taiwan University Hospital, Taipei, Taiwan; 5 Department of Nutrition, School of Public Health, Harvard University, Boston, Massachusetts, United States of America; Duke Clinical Research Institute, United States of America

## Abstract

**Background:**

Our aim was to prospectively investigate the association between carotid artery intima-media thickness (IMT) as well as carotid plaque and incidence of coronary heart disease (CHD) and stroke in Chinese, among whom data are limited.

**Methods and Findings:**

We conducted a community-based cohort study composed of 2190 participants free of cardiovascular disease at baseline in one community. During a median 10.5-year follow up, we documented 68 new cases of coronary heart disease and 94 cases of stroke. The multivariate relative risks (RRs) associated with a change of 1 standard deviation of maximal common carotid IMT were 1.38 (95% confidence interval [CI], 1.12–1.70) for CHD and 1.47 (95% CI, 1.28–1.69) for stroke. The corresponding RRs with internal carotid IMT were 1.47 (95% CI, 1.21–1.79) for CHD and 1.52 (95% CI, 1.31–1.76) for stroke. Carotid plaque measured by the degree of diameter stenosis was also significantly associated with increased risk of CHD (p for trend<0.0001) and stroke (p for trend<0.0001). However, these associations were largely attenuated when adjusting for IMT measurements.

**Conclusions:**

This prospective study indicates a significant association between carotid IMT and incidence of CHD and stroke in Chinese adults. These measurements may be useful for cardiovascular risk assessment and stratification in Chinese.

## Introduction

Cardiovascular disease remains the leading cause of death worldwide. International studies have demonstrated that Asian and Western populations have different patterns of cardiovascular disease events[1], [2]. In Western countries, coronary heart disease (CHD) is more common than stroke, whereas in Asian-Pacific countries, stroke outnumbers CHD [3]. Because of ethnic differences in the atherosclerosis process[4], it is important to investigate the role of subclincial atherosclerosis in the development of CHD and stroke in Asian populations.

Intima-media thickness (IMT) measurements of the common carotid artery (CCA) and internal carotid artery (ICA) are considered as useful indicators of carotid atherosclerosis[5]–[10]. Previous studies have only focused on common measurements of carotid arteries and did not discriminate the potential differences between CCA and ICA[10], [11]. Evidence showed that CCA and ICA narrowing may reflect different pathophysiology and thus may be differentially related to cardiovascular events.[12]. Moreover, the carotid plaque severity has also been associated with increased risk of cardiovascular disease [11], [13]–[16]. Therefore, we conducted a prospective community-based cohort study to examine the associations of carotid artery IMT and carotid plaque with incidence of CHD and stroke in Chinese in Taiwan.

## Results

Higher IMT was associated with higher prevalence of obesity, hypertension, and the metabolic syndrome (table 1). The correlations between IMT and other risk factors ranged from 0.07 for triglycerides to 0.20 for systolic blood pressure (Table 2). The IMT measurements were correlated with the carotid plaque score (0.34–0.37). The carotid plaque score was minimally correlated with other risk factors, ranging −0.06 for body mass index to 0.06 for serum cholesterol.

**Table 1:** Characteristics of the 2190 study participants at enrollment according to quartiles of maximal ICA intima media thickness and plaque stenosis severity measurements.

ICA	1	2	3	4		Plaque	0	1	2	> = 3	
Range	<0.55	0.55–0.70	0.70–0.85	> = 0.85							
	N = 560	N = 796	N = 416	N = 418			N = 1876	N = 93	N = 70	N = 151	
	(%)	(%)	(%)	(%)	P		(%)	(%)	(%)	(%)	P
Gender					<.0001	Gender					<.0001
Men	34.8	41.1	52.4	58.1		Men	43.0	51.6	51.4	61.6	
Women	65.2	58.9	47.6	41.9		Women	57.0	48.4	48.6	38.4	
Current smoker (yes)	21.1	31.8	38.2	48.8	<.0001	Current smoker (yes)	31.5	36.6	47.1	50.3	<.0001
Alcohol drinking (yes)	23.0	28.8	32.7	31.8	0.0029	Alcohol drinking (yes)	28.1	25.8	27.1	37.1	0.11
Married status					<.0001	Married status				<.0001	
Single	1.4	2.6	4.1	2.6		Single	2.4	2.2	10.0	2.0	
Lived with spouse	92.5	87.4	84.3	79.3		Lived with spouse	88.3	79.6	64.3	79.5	
Divorced	6.1	10.0	11.6	18.0		Divorced	9.3	18.3	25.7	18.5	
Education level					0.25	Education level					0.23
<9 yr	91.8	93.2	93.8	95.0		<9 yr	92.9	96.8	94.3	96.0	
> = 9 yr	8.2	6.8	6.3	5.0		> = 9 yr	7.1	3.2	5.7	4.0	
Job status					<.0001	Job status					<.0001
No job	36.4	48.0	50.7	61.5		No job	44.5	68.8	71.4	69.5	
Labor work	38.0	34.9	33.2	27.5		Labor work	36.0	22.6	21.4	21.9	
Professional	25.5	17.1	16.1	11.0		Professional	19.5	8.6	7.1	8.6	
Regular exercise habit	12.1	15.6	16.6	19.4	0.019	Regular exercise habit	14.8	16.1	27.1	19.9	0.0181
Family history of CHD	12.0	10.1	8.2	9.1	0.23	Family history of CHD	10.7	6.5	4.3	6.6	0.08
Hypertension	14.0	25.9	32.2	44.1	<.0001	Hypertension	25.0	35.5	40.0	48.3	<.0001
Diabetes	9.7	10.8	14.2	18.1	<.0001	Diabetes	11.4	24.7	15.7	17.9	0.0002
Metabolic syndrome	16.0	22.2	29.2	31.9	<.0001	Metabolic syndrome	22.5	29.0	37.1	30.2	0.0041
	Mean						Mean				
CCA+ICA, IMT	0.51	0.66	0.81	1.09	<.0001	CCA+ICA, IMT	0.68	0.87	0.99	1.20	<.0001
CCA, IMT	0.53	0.67	0.82	1.08	<.0001	CCA, IMT	0.68	0.88	1.00	1.25	<.0001
ICA, IMT	0.49	0.65	0.80	1.10	<.0001	ICA, IMT	0.67	0.86	0.98	1.14	<.0001
Carotid plaque	0.01	0.08	0.34	2.23	<.0001	Carotid plaque	0	1	2	6.0	<.0001
Age	46.5	52.3	57.6	64.2	<.0001	Age	52.2	62.4	65.6	66.6	<.0001
BMI, baseline	23.3	23.7	24.0	23.5	0.0077	BMI, baseline	23.7	23.5	22.4	22.8	0.0003
Waist	80.4	82.9	85.1	85.5	<.0001	Waist	83.0	84.0	84.2	84.3	<.0001
Systolic BP	116.8	122.8	127.6	134.7	<.0001	Systolic BP	122.9	127.8	133.0	137.5	<.0001
Diastolic BP	74.6	77.1	78.2	78.7	<.0001	Diastolic BP	76.8	78.3	78.2	78.1	0.18
Cholesterol	191.4	198.5	204.2	204.7	<.0001	Cholesterol	197.3	205.6	208.9	210.6	0.0004
Triglyceride	112.5	129.4	130.7	133.8	0.0007	Triglyceride	124.8	136.7	139.5	130.9	0.32
HDL-Cholesterol	48.8	47.2	47.0	45.2	<.0001	HDL-Cholesterol	47.4	45.2	44.5	47.6	0.09
LDL-Cholesterol	130.6	138.4	144.9	147.3	<.0001	LDL-Cholesterol	137.5	148.3	150.7	150.9	<.0001
Glucose	105.5	108.0	111.4	116.6	<.0001	Glucose	109.0	116.7	111.5	112.8	0.05

Abbreviation: CCA, common carotid artery, ICA, internal carotid artery, IMT, intima-media thickness.

**Table 2:** Adjusted Spearman Correlation coefficients of carotid-artery intima-media thickness, plaque scores and various atherosclerotic risk factors.

	CCA, IMT	ICA, IMT	Plaque	BMI	SBP	DBP	Cholesterol	TG	HDL	LDL	Glucose
CCA, IMT		0.77	0.37	0.12	0.21	0.14	0.09	0.07	−0.07	0.11	0.06
ICA, IMT			0.34	0.11	0.18	0.13	0.08	0.07	−0.09	0.10	0.07
CCA & ICA, IMT	0.94	0.94	0.36	0.12	0.20	0.14	0.09	0.07	−0.08	0.11	0.07
Carotid plaque				−0.06	0.05	0.01	0.06	0.03	−0.01	0.06	0.00
BMI					0.30	0.30	0.16	0.36	−0.35	0.25	0.21
SBP						0.72	0.10	0.24	−0.15	0.14	0.11
DBP							0.12	0.22	−0.13	0.15	0.11
Cholesterol								0.27	0.10	0.94	0.14
TG									−0.48	0.39	0.23
HDL-cholesterol										−0.16	−0.17
LDL-cholesterol											0.19

Abbreviation: CCA, common carotid artery; ICA, internal carotid artery; IMT, intima-media thickness; BMI, body mass index; SBP, systolic blood pressure; DBP, diastolic blood pressure; TG: triglycerides; HDL, high density lipoprotein; LDL, low density lipoprotein.

The incidence of CHD and stroke events increased progressively with increasing quartiles for IMT measurements (Table 3). After multivariate adjustments, the relative risks (RRs) associated with a change of 1 standard deviation of maximal common carotid IMT were 1.38 (95% confidence interval [CI], 1.12–1.70) for CHD and 1.47 (95% CI, 1.28–1.69) for stroke. Internal carotid IMT had a slightly higher risk for CHD and stroke: the corresponding RRs with 1 standard deviation of maximal internal carotid IMT were 1.47 (95% CI, 1.21–1.79) for CHD and 1.52 (95% CI, 1.31–1.76) for stroke. Carotid plaque was also significantly associated with cardiovascular events: the multivariate RRs with 1 unit increment in carotid plaque were 1.15 (95% CI, 1.07–1.24) for CHD and 1.11 (95% CI, 1.05–1.18) for stroke events. Analyses based on the quartiles of common and internal carotid IMT and carotid plaque showed similar results as those from the continuous analyses.

**Table 3:** Median level, incident rates, relative risks and 95% confidence intervals of coronary heart disease and stroke in the study participants according to maximal CCA IMT quartile and carotid plaque.

**Maximal CCA IMT**															
Quartile	1	2			3			4							
Median (mm)	0.55	0.65			0.80			1.00							
**Coronary Heart Disease**															
Rate/1,000	1.5	1.8			3.8			8.2			P, trend	Per 1 SD (0.26 mm) increase			P
Relative risk and 95% CI															
Model 1	1	0.87	0.35	2.19	1.42	0.62	3.26	2.47	1.11	5.47	0.006	1.41	1.17	1.70	0.0003
Model 2	1	0.78	0.31	1.96	1.26	0.55	2.90	2.08	0.94	4.63	0.018	1.41	1.15	1.72	0.001
Model 3	1	0.65	0.25	1.70	1.08	0.46	2.51	1.78	0.79	3.97	0.037	1.38	1.13	1.70	0.002
Model 4	1	0.66	0.25	1.74	1.08	0.47	2.52	1.75	0.78	3.94	0.045	1.38	1.12	1.70	0.003
**Stroke**															
Rate/1,000	1.5	3.7			5.3			11.1			P, trend	Per 1 SD (0.26 mm) increase			P
Relative risk and 95% CI															
Model 1	1	1.66	0.75	3.67	1.52	0.70	3.33	2.36	1.12	4.97	0.023	1.54	1.35	1.75	<.0001
Model 2	1	1.69	0.76	3.75	1.53	0.70	3.36	2.50	1.19	5.28	0.014	1.61	1.41	1.83	<.0001
Model 3	1	1.68	0.73	3.87	1.57	0.70	3.54	2.27	1.04	4.96	0.046	1.49	1.30	1.70	<.0001
Model 4	1	1.66	0.72	3.83	1.57	0.70	3.54	2.21	1.01	4.85	0.055	1.47	1.28	1.69	<.0001
**Carotid Plaque**															
Score	0	1			2			> = 3							
Coronary Heart Disease															
Rate/1,000	2.4	4.9			10.1			13.9			P, trend	Per 1 increase			P
Relative risk and 95% CI															
Model 1	1	1.76	0.62	5.03	2.97	1.20	7.35	3.59	1.85	6.95	<.0001	1.11	1.04	1.18	0.001
Model 2	1	1.77	0.62	5.11	2.99	1.16	7.70	3.95	2.02	7.71	<.0001	1.13	1.05	1.20	0.001
Model 3	1	1.69	0.59	4.88	2.61	0.99	6.88	3.91	1.98	7.71	<.0001	1.15	1.06	1.23	0.000
Model 4	1	1.67	0.58	4.82	2.67	1.01	7.11	3.85	1.91	7.74	<.0001	1.15	1.07	1.24	0.000
Stroke															
Rate/1,000	3.5	6.2			11.8			17.6			P, trend	Per 1 increase			P
Relative risk and 95% CI															
Model 1	1	1.07	0.43	2.68	1.53	0.68	3.42	2.19	1.28	3.76	0.005	1.13	1.08	1.18	<.0001
Model 2	1	1.21	0.48	3.05	1.40	0.61	3.21	2.39	1.38	4.13	0.003	1.15	1.09	1.20	<.0001
Model 3	1	1.16	0.46	2.96	1.32	0.56	3.14	2.09	1.18	3.68	0.014	1.11	1.05	1.18	0.000
Model 4	1	1.18	0.46	3.01	1.27	0.53	3.02	1.96	1.10	3.49	0.029	1.11	1.05	1.18	0.001

Model 1: adjusted for age groups (35–44, 45–54, 55–64, 65–74, > = 75 years old) and gender.

Model 2: Model 1 plus body mass index (<18, 18 to 20.9, 21 to 22.9, 23 to 24.9, or > = 25 kg/m^2^), alcohol intake (nondrinker/regular), exercise (yes/no), marital status (single, married or divorced), education level (<9 years, ≥9 years), occupation (no work, manual work, or professional), and family history of coronary heart disease (yes/no).

Model 3: Model 2, adding baseline hypertension, diabetes, continuous HDL-C and LDL-C variables.

Model 4: Model 3, adding metabolic syndrome (yes/no).

In multivariate analyses including both IMT and carotid plaque score in the same models (Table 4), the association of common carotid IMT with CHD becomes nonsignificant (multivariate RR, 1.16, 95% CI, 0.86–1.57), but its association with stroke risk remained significant (RR, 1.48, 95% CI, 1.21–1.80). The internal carotid IMT remained significant for both CHD and stroke. However, the relative risk of carotid plaque diminished appreciably and became nonsignificant after adjusting for IMT in the models.

**Table 4:** Relative risk and 95% confidence intervals for jointed analysis of per 1 SD increase in IMT and 1 score increase in plaque score for CHD and stroke.

Coronary Heart Disease								
	Relative risk and 95% CI			*P*	Relative risk and 95% CI			*P*
	CCA				Plaque			
Model 1	1.31	1.00	1.71	0.05	1.04	0.95	1.14	0.39
Model 2	1.25	0.95	1.64	0.11	1.07	0.98	1.17	0.15
Model 3	1.18	0.88	1.59	0.27	1.10	0.99	1.22	0.09
Model 4	1.16	0.86	1.57	0.34	1.11	0.99	1.24	0.07
	ICA				Plaque			
Model 1	1.41	1.13	1.77	0.003	1.04	0.96	1.13	0.36
Model 2	1.40	1.11	1.76	0.004	1.06	0.97	1.15	0.18
Model 3	1.34	1.05	1.70	0.019	1.08	0.98	1.18	0.11
Model 4	1.33	1.04	1.69	0.023	1.08	0.99	1.19	0.10
Stroke								
	CCA				Plaque			
Model 1	1.47	1.22	1.78	<.0001	1.02	0.95	1.10	0.55
Model 2	1.53	1.26	1.85	<.0001	1.03	0.95	1.11	0.47
Model 3	1.49	1.23	1.81	<.0001	1.00	0.92	1.08	0.94
Model 4	1.48	1.21	1.80	0.000	1.00	0.92	1.08	0.95
	ICA				Plaque			
Model 1	1.42	1.19	1.70	<.0001	1.05	0.99	1.12	0.13
Model 2	1.44	1.21	1.73	<.0001	1.06	0.99	1.13	0.11
Model 3	1.50	1.24	1.82	<.0001	1.01	0.94	1.09	0.79
Model 4	1.50	1.23	1.81	<.0001	1.01	0.93	1.09	0.83

Model 1: adjusted for age groups (35–44, 45–54, 55–64, 65–74, > = 75 years old) and gender.

Model 2: Model 1 plus body mass index (<18, 18 to 20.9, 21 to 22.9, 23 to 24.9, or > = 25 kg/m^2^), alcohol intake (nondrinker/regular), exercise (yes/no), marital status(single, married or divorced), education level ((<9 years, ≥9 years), occupation (no work, manual work, or professional), and family history of coronary heart disease (yes/no).

Model 3: Model 2, adding baseline hypertension, diabetes, continuous HDL-C and LDL-C variables.

Model 4: Model 3, adding metabolic syndrome (yes/no).

In stratified analyses, common and internal carotid IMT predicted CHD risk in most subgroup analyses defined by cardiovascular risk factors, and only the tests for interaction between IMT and diabetes were statistically significant (*P* for interaction = 0.03 for CCA) (data not shown). Common and internal carotid IMT was also significantly associated with the risk of stroke in most subgroups (all *P* for interaction >0.05).

Adding IMT provided only a slight improvement in predicting the risk of CHD and stroke beyond the standard risk factors (Table 5). First, the increase in the AUC (from 0.787 to 0.798 for CHD, from 0.822 to 0.829 for stroke) reached a borderline significant level after adding IMT to the models with traditional risk factors for CHD. Second, adding IMT information resulted in a slightly better integrated discrimination improvement (IDI) for stroke (IDI = 0.022, *P* = .011) and CHD (IDI = 0.0035, *P* = .09). These findings showed non-significant improvement by adding IMT information.

**Table 5:** Summary statistics comparing risk prediction for the models without and with IMT for the risk of CHD and stroke.

	AUC	P	IDI*	P	NRI†	P
CHD		0.10	0.004	0.09	0.086	0.09
Without IMT*	0.787					
With IMT	0.798					
Stroke		0.28	0.022	0.011	0.079	0.12
Without IMT	0.822					
With IMT	0.829					

Abbreviation: AUC, area under receiver operative characteristic curve; CHD, coronary heart disease; IMT, intima-media thickness; IDI, Integrated discrimination improvement; NRI, Net reclassification improvement.

* Integrated discrimination improvement.

† Net reclassification improvement with a priori risk categories according to (0–5%, 5–10%, 10–20%, and > = 20%).

## Discussion

The results of this large prospective study of middle-aged and older Chinese indicate that elevated carotid IMT measurements significantly predict an increase risk of CHD and stroke in healthy Chinese, independent of other cardiovascular risk factors. These data provide useful information on the potential utility of IMT measurements and carotid sonography in screening subclinical cardiovascular disease in populations with relatively low CHD but high stroke risk.

Carotid sonography has been recommended as a screening tool for future cardiovascular events among high-risk populations, such as elderly adults[17], type 2 diabetics[18] or stable CHD patients[19]. Updated consensus has proposed the standards for measurement of IMT and plaque in the carotid artery[20], [21]. However, there is a disagreement regarding the use of these measurements as screening tool in the general population[20]. And it has been recommended that more data need to be collected for different ethnic groups. Our study has partially filled this gap.

Carotid artery IMT and plaque stenosis, as markers of subclincial atherosclerosis, reflect not only early atherosclerosis but also compensatory enlargement with medial hypertrophy as a result of smooth muscle cell proliferation reactions[20], [22]. Because atherosclerosis develops in men at an earlier stage, carotid IMT is greater in men than in women. Common and internal carotid artery IMT progression has been related to several cardiovascular risk factors including smoking, hypertension and hyperglycemia [23]. In the stiffer arteries such as ICA, systolic blood pulse is augmented by fast travel of pulse wave and the blood flow velocity is reduced in diastole, further accelerating lipid deposition and local inflammation and results in increasing thickness of intima medial layers in ICA[24].

Clinical observations suggested that the higher blood pressure and vascular wall shear stress on the left carotid artery resulted in higher common carotid IMT on left side[25], [26]; however, the side difference was only limited to CCA and did not affect the prediction of subsequent cardiovascular events[27]. Our study did not show differential effects of measurements from different sides on CVD risk. Also, there were no appreciable gender, age, smoking, hypertension, obesity and hyperlipidemia differences in the role of IMT for predicting risk of CHD and stroke. Nevertheless, our findings showed a slightly higher risk for CHD and stroke for ICA than for CCA, but the difference was small.

Several cohort studies have explored the association between carotid artery IMT and the incidence of CHD and stroke in Western populations[5], [6], [10], [28], [29]. After one year follow-up among 1257 middle-aged Finnish men, common carotid IMT was associated a 3.3-fold increased risk for CHD event[28]. In the Rotterdam Elderly Study including 7983 participants older than 55 years and follow up for 6 years, common carotid IMT was a significant predictor for stroke and CHD[6], [30]. Chambless and colleagues demonstrated that the combined CCA and ICA measurements were significant predictors of CHD among 15792 middle-aged adults in the Atherosclerosis Risk in Community cohort[29]. In another study based on 5858 older adults (65 years of age or older) and 6 year of follow-up , O'Leary and colleagues demonstrated that both common and internal carotid IMT measurements were significant predictors of CHD and stroke[10].

Few studies have examined the role of carotid IMT in predicting CVD events in Asian populations[31]–[34]. In a study of Japanese diabetic patients, carotid IMT was associated with increased CHD events during a 3-year follow up [32]. Among 298 elderly Japanese (older than 75 years, average 80 years), carotid IMT was associated with increased cardiovascular death and total mortality during 3 years of follow-up[34]. Our study provided strong evidence that carotid IMT significantly predicts CHD and stroke in a community-based healthy Chinese population. Furthermore, our findings were compatible with a recent meta-analysis results which showed one standard deviation of IMT difference increased a 1.26-fold risk for CHD and a 1.32-fold risk for stroke [35].

Carotid plaque provided additional information for cardiovascular risk prediction because the plaque score reflects the severity of irregular morphology and lumen narrowing[14], [36]. Carotid plaque was reported to be associated with local inflammation and biomechanical stress[36] and was considered as a marker of advanced atherosclerosis. Furthermore, ethnic variation in carotid plaque severity has been demonstrated and African Americans men appear to have appreciably lower carotid plaque than white men.[37] Cross-sectional studies found that carotid plaque was significantly associated with prevalence of CHD[38], and the prospective cohort data suggested that carotid plaque predicted future risk of ischemic stroke among 1939 U.S. adults[15] and among 1289 elderly Japanese men[11]. Our findings suggested that the carotid plaque was significantly associated with risk of CHD and stroke, but the association was largely explained by IMT.

To our knowledge, this is the first extensive investigation of carotid artery structure and risk of CHD and stroke among Chinese. Because of the prospective cohort design, the baseline measurements of our cohort members were unlikely to be affected by disease status. Furthermore, the use of a community-based population could reduce the possibility of selection bias. We also included important covariates including socioeconomic status, lifestyle factors, and well-established CVD risk factors including hypertension, diabetes, blood lipid profiles and the metabolic syndrome. Adjustment for these variables did not diminish the role of IMT in predicting CHD or stroke.

Our study had several potential limitations. First, the number of incident cases of CHD and stroke events was relatively small, even with more than a decade's follow up, which would reduce the power to detect the subtle differences between common and internal carotid artery IMT and make the relative risk estimation unstable. However, the 95% confidence intervals for the estimated relative risks were narrow and tests for linear trends were significant for our exposure variables. Second, we did not measure functional parameters such as resistance index, which might be useful for further risk stratification. In addition, there was no formal comparison of our results to those from other racial/ethnic groups. Nonetheless, our findings added to the existing literature about the role of carotid atherosclerosis for further cardiovascular risk.

In conclusion, we demonstrate that IMT and carotid plaque were associated the risks of CHD and stroke among Chinese. Because of only moderate correlation coefficients between carotid artery measurements and traditional vascular risk factors, the carotid artery measurements especially IMT can be useful for comprehensive evaluation of cardiovascular risk in Asian populations.

## Methods

### Study Design and Study Participants

Details of this cohort study have been published previously[39]–[41]. Briefly, the Chin-Shan Community Cardiovascular Cohort Study (CCCC) began in 1990 by recruiting 1703 men and 1899 women of Chinese ethnicity aged 35 years old and above from the Chin-Shan township, 30 km north of metropolitan Taipei, Taiwan. Lifestyle information and medical conditions were assessed by interview questionnaires at 2-year cycles for the initial 6 years, and the validity and reproducibility of these data and anthropometric measurements have been reported in detail elsewhere[42]. Two thousand and two hundred forty-four participants had complete carotid ultrasonography measurements in 1994–1995. After excluding those with previous history of cardiac disease (n = 16) and cerebrovascular disease (n = 38), a total of 2190 participants were included in this study. For cohort follow up from 1994 to the end of 2005 (a total of 20,102.7 person-years, median 10.5 years, inter-quartile range: 9.5–10.6 years), we documented 68 incident cases of CHD, 94 incident cases of stroke (including 79 cases of ischemic and unclassified type and 15 cases of hemorrhagic type). Incident CHD cases were defined by nonfatal myocardial infarction, fatal coronary heart disease and hospitalization due to percutaneous coronary intervention and coronary bypass surgery. Deaths were identified from official certificate documents, further verified by house-to-house visits. Fatal CHD was considered to have occurred if fatal myocardial infarction was confirmed by hospital records or if CHD was listed as the cause of death on the death certificate as the underlying and most plausible cause of death, or if evidence of previous CHD was available. Incident stroke cases were ascertained according to the following criteria: a sudden neurological symptom of vascular origin that lasted longer than 24 hours, with supporting evidence from the image study. Transient ischemic attacks were not included in this study. The cases were confirmed by internists. The National Taiwan University College of Public Health Committee Review Board approved the study protocol. The participants gave verbal informed consent due to minimal harm to the participants and the requirement to obtain a signed consent form in this non-invasive observational study was waived.

### Measurement of biochemical markers

The procedures of blood sample collection were reported elsewhere [43], [44]. Briefly, all venous blood samples drawn after a 12-hour overnight fast were immediately refrigerated and transported within 6 hours to the National Taiwan University Hospital. Serum samples were then stored at −70°C before batch assay for levels of total cholesterol, triglycerides, and high density lipoprotein cholesterol (HDL-C). Standard enzymatic tests for serum cholesterol and triglycerides were used (Merck 14354 and 14366, Germany, respectively). HDL-C levels were measured in supernatants after the precipitation of specimens with magnesium chloride phosphotungstate reagents (Merck 14993). LDL-C concentrations were calculated as total cholesterol minus cholesterol in the supernatant by precipitation method (Merck 14992)[45].

### Carotid artery ultrasonographic measurements

The measurements of IMT were obtained by using a Hewlett-Packard SONO 1500 ultrasound system, equipped with a 7.5 MHz real-time B-mode scanner to examine the patient's carotid arteries. Patients were asked to lie supine with the neck extended in a slightly lateral rotation. Then we scanned the carotid artery and found the lumen of the carotid artery beneath the surface of the neck. We defined IMT as the distance from the front edge of the first echogenic line (lumen-intima interface) to the front edge of the second line (media-adventitia interface) in the far wall of the vessel. We performed the same procedures on the other side of the neck. The maximal IMT was defined by averaging maximal measurement on both sides[46]. The inter-observer reliability of these measurements using the inter-rater and intra-rater correlation reliability ranged from 0.70 to 0.93[47].

The quantification of carotid plaque was described elsewhere [39], [47]. Briefly, carotid artery segment, including proximal CCA (>20 mm proximal to the bulb bifurcation), distal CCA, bulb, ICA, and external carotid artery were examined bilaterally. A grade was assigned to each chosen segment: grade 0 for normal or no observable plaque; grade 1 for one small plaque with diameter stenosis <30%; grade 2 for one medium plaque with 30% to 49% diameter stenosis or multiple small plaques; grade 3 for one large plaque with 50% to 99% diameter stenosis or multiple plaques with at least one medium plaque; and grade 4 for 100% occlusion. The carotid plaques on the proximal and distal CCA, the bulb, and the internal and external carotid arteries, were measured and given numerical values; measurements were tallied and an individual was assigned a composite score for the severity of carotid plaque. Reproducibility of plaque grade scores was good, with a kappa value of 0.70[47].

### Statistical analysis

Participants were categorized into quartiles of common and internal carotid artery IMT measurements. Continuous variables were presented by mean (standard deviation) or median levels, and categorical data were presented in contingency tables. Correlations between baseline IMT and other cardiovascular risk factors were estimated by Spearman's partial correlation coefficients adjusted for age and gender.

Incidence rates of CHD and stroke were calculated by dividing the number of cases by the person-years of follow up for each quartile of IMT. Relative risk of CHD and stroke was calculated by dividing the incidence rate of each quartile by the rate in the first quartile. In addition, we estimated the relative risks associated with a change of one standard deviation in the IMT. We used Cox proportional-hazards models to adjust for potential confounding variables. We specified four models to estimate relative risk of CHD and stroke. In model 1, we adjusted for age groups (35–44, 45–54, 55–64, 65–74, > = 75 years old) and gender. In Model 2, we additionally adjusted for body mass index (<18, 18 to 20.9, 21 to 22.9, 23 to 24.9, or > = 25 kg/m^2^) and lifestyle factors, including alcohol intake (nondrinker/current), smoking, (yes/no) and exercise(yes/no), as well as socioeconomic status, including marital status(single, married, or divorced), educational level (<9 years, ≥9 years), occupation (no work, manual work, or professional), and family history of CHD (yes/no). In Model 3, we adjusted further for the presence or absence of hypertension and diabetes and continuous variables including LDL and HDL cholesterol levels. In model 4, we further adjusted for the presence or absence of the metabolic syndrome defined by the NCEP ATP III criteria[48]. To test for linear trend across IMT quartiles, we used the median IMT value for each quartile. The goodness of fit for each model was tested by the Hosmer and Lemeshow test[49]. We conducted stratified analyses to evaluate a potential effect modification by baseline hypertension (absence or presence), diabetes (absence or presence), total cholesterol, and body mass index using median values as the cutoffs.

We compared the performance of the models without and with CCA IMT information using the area under the receiver operating characteristic curve (AUC). The curve is a graph of sensitivity versus 1-specificity (or false-positive rate) for various cutoff definitions of a positive diagnostic test result[50]. Statistical differences in the AUCs were compared using the method of DeLong et al[51]. However, the AUC value is not the best discriminatory statistics for prediction power[52]–[54]. Therefore, we provided several additional statistics, including integrated discrimination improvement (IDI) and net reclassification improvement (NRI) [53] for the comparison of nested models with and without IMT. The IDI is considered the difference between improvement in average sensitivity and any potential increase in average ‘one minus specificity’[53], and is estimated as the difference in Yates discrimination slopes between the nested models[55], [56]. The reclassification table as a tool for comparing the models was suggested by Ridker and colleagues[54]. Pencina and colleagues constructed the reclassification tables and developed a NRI (net reclassification improvement) statistic according to a sum of differences between the ‘upward’ movement in categories for event subjects and the “downward’ movement in those for nonevent subjects[53]. A priori risk categories were defined as 0–5%, 5–10%, 10–20%, and > = 20%.

All statistical tests were two-tailed with a type I error of 0.05, and *P* values<0.05 were considered statistically significant. Analyses were performed with SAS version 9.1 (SAS Institute, Cary, NC) and Stata version 9.1 (Stata Corporation, College Station, Texas).
